# Sensor-Based Smart Clothing for Women’s Menopause Transition Monitoring

**DOI:** 10.3390/s20041093

**Published:** 2020-02-17

**Authors:** Jie Luo, Aihua Mao, Zhongwen Zeng

**Affiliations:** 1 School of Fine Art and Artistic Design, Guangzhou University, Guangzhou 510006, China; jieluo@gzhu.edu.cn; 2 School of Computer Science & Engineering, South China University of Technology, Guangzhou 510006, China; 3 Donghuzhou Primary School, Guangzhou 511400, China; ZZW1543901322@163.com

**Keywords:** menopause transition, smart clothing, sensors, detection algorithm

## Abstract

Aging women usually experience menopause and currently there is no single diagnosing highly-sensitive and -specific test for recognizing menopause. For most employed women at their perimenopause age it is not convenient to visit a clinic for the hormone test, which lasts for consecutive days. This paper develops a suit of sensor-based smart clothing used for home-based and ambulatory health monitoring for women’s menopause transition. Firstly, a survey analysis is conducted to determine the biological signals measured by sensors for indicating the symptoms of menopausal transition and also the body areas with salient symptoms to implant the sensors on the clothing. Then, the smart clothing is designed with a set of temperature and relative humidity sensors on different locations and with a microcontroller to transmit the measured data to the computer. With the smoothed data as input, a new detection algorithm for hot flashes is proposed by recognition of the concurrent occurrence of heat and sweating rise/down, and can figure out the frequency, intensity, and duration—triple dimension information of a hot flash, which is helpful to achieve precise diagnosis for menopausal transition. The smart clothing and the detection algorithm are verified by involving a group of women subjects to participate in a hot flash monitoring experiment. The experimental results show that this smart clothing monitoring system can effectively measure the skin temperature and relative humidity data and work out the frequency, duration, and intensity information of a hot flash pertaining in different body areas for individuals, which are accordant with the practice reported by the subjects.

## 1. Introduction

Menopause is a universal biological event among aging women and happens to normal women at a mean age of 51; reports indicate that, 95% of women become menopausal between 45 and 55 years of age [1]. Several women below 45 years may experience an early menopause due to primary ovarian insufficiency or gynecological interventions [2]. Menopausal transition, which is also termed perimenopause, refers to the period linking the women’s reproductive and menopausal years. This transition greatly varies for individuals but may extend for 5–10 years before menopause [3]. During menopause transition, women often have the etiology of symptoms, such as hot flashes, night sweats, sleeping disturbances, headaches, low mood or anxiety, reduced muscle mass, vulvovaginal atrophy, and sexual dysfunction [4,5,6]. Several symptoms often happen simultaneously and can be unbearable. Such symptoms can intervene with women’s daily activities and sleep quality and may lead to their difficulties. The duration of menopausal transition is different for each woman but it commonly lasts for around four years [7]. Therefore, research on the recognition of menopausal transition is significant to clinicians, who make the decisions to provide treatment for the relief of symptoms.

Currently, no single diagnosing test is highly sensitive and specific for recognizing menopause. The best way to recognize natural menopause is to observe amenorrhea for 12 consecutive months retrospectively, and the best predicted actual transition to menopause within 4 years is amenorrhea between 3 and 12 months’ duration [8]. For clinical situations, the level of follicle-stimulating hormone is generally monitored to diagnose menopausal transition [9]. However, the individual hormone levels may fluctuate greatly at different times [10]. In particular, a woman’s hormonal levels could be high for one day but low for the next day. Thus, testing hormone level remains an unreliable indicator to diagnose menopause. In addition, most employed women at their perimenopause age lack the time to visit the clinic for the hormone test, which is administered for consecutive days. A sensitive and convenient testing/monitoring device and method should be utilized to assist menopausal diagnosing.

The increasing popularity of wearable monitoring systems has promoted smart textile or smart clothing as the next generation of wearable technology, which is intuitive for developing various applications of daily dressing for intelligent healthcare. Wearable devices enable early detection through long-term trend analysis in daily life, particularly for the elderly. Currently, substantial progress has been achieved in the field of wearable devices for intelligent care. It mainly includes: (1) smart clothing for detecting physiological signals, such as, heart rate, breathing rate and muscle tension [11,12], respiratory frequency and motion [13]; (2) smart accessories, like smart tiles for fall detection [14], smart eyeglass frames for monitoring temporalis muscle activity [15]; and (3) textile-based sensors, like textile antenna for electromagnetic energy harvesting [16] and portable tri-axis sensors for fall monitoring [17]. Recently, Fang et al. proposed design method and application of integrating interactive clothing and cyber–physical human centric system in smart homes [18,19]. Smart clothing embedded with noninvasive sensors is a prospective solution for home-based and ambulatory health monitoring because clothing is in direct contact with the human’s skin for the most time.

When specific sensors, such as thermal sensors, electrocardiography sensors, myocardial sensors, blood oxygen sensors, and electroencephalographic sensors, are attached, smart clothing can measure various biological information of the human body, such as body skin temperature, humidity, heat rhythms, and blood leakage [20]. Thus, the etiology of symptoms of women’s menopausal transition must theoretically be analyzed to determine the most substantial biological signals corresponding to the salient symptoms. The appropriate sensors can then be selected to design a smart clothing system for monitoring women’s menopausal transition. In this paper, we develop a suit of sensor-based smart clothing for convenient and sensitive monitoring of menopausal transition particularly for employed women. When this clothing is worn, the attached sensors automatically gather skin temperature and relative humidity data from different body areas. These data are transmitted to the personal computer (PC) ender where they are processed and input into a new proposed algorithm for detecting hot flashes. Experimental analysis with a group of subjects shows that the smart clothing can successfully collect biological data. Moreover, the detection algorithm can output useful detection information including frequency, duration, and intensity of hot flashes, whereas previous work was merely concerned with frequency. These detection results offer precise diagnosis for menopausal transition and are consistent with the practical symptoms of the involved subjects. The main contributions of this study include the following:

(1) Biological signals related to the etiology of symptoms of women’s menopausal transition and body areas with salient symptoms are determined through a survey analysis.

(2) A suit of sensor-based smart clothing system that attaches temperature and relative humidity sensors on different locations to collect skin biological signals and a microcontroller connecting all sensors to transmit the data to the computer are designed.

(3) A new trinity (frequency–duration–intensity) for a hot flash detection algorithm, which takes the refined sensor data in multiple body areas as input and calculates frequency, intensity, and duration, is proposed by analyzing the biological inference between heat and sweating rise/down and hot flash.

## 2. Survey Analysis of Menopausal Requirements for Smart Clothing Design

Menopausal symptoms can affect the lives of elderly and some aging women below 45 years. Over 30 symptoms are associated with menopause, and hot flashes are the most salient [21]. Griffiths et al. explored the menopausal symptom experiences among a group of women aged between 45 and 55 years [22]. They found that 40% of the subjects experienced hot flashes, that is, a sudden sense of heat spreading throughout the body and flushing of face and chest with sweating and occasional chills. Other symptoms include sleep disturbances (56%), night sweats (43%), tiredness (53%), mood swings (35%), and palpitations (20%). Approximately 80% women experience hot flashes for 5 years and above due to decreasing levels of estrogen [23]. Moreover, hot flashes are commonly accompanied by night sweats, which refer to episodes of drenching sweats at night. Both are vasomotor symptoms and are the most common physical changes experienced by 70% of women in Europe and North America [24]. In order to determine the menopausal requirements for the smart clothing design, which could investigate some representatives to reveal the needs of the majority [25], we designed a survey questionnaire based on these findings and the comments by the experts in gynecology. We subsequently interviewed 100 employed women aged between 40–60 years on the public sites/plazas in Guangzhou city to complete the questionnaire During the survey, the participants were informed of the background of this research firstly and then finished the choice questions by ticking the right answers. The participants held different educational degrees. As mentioned by Delavar et al. [26], there was no relationship between marital status, education, occupation, and economic situation with the menopausal symptoms, thus we categorize the occupation of participants in terms of indoor and outdoor jobs. Totally, 82% of them are engaged in indoor jobs and 18% of them are engaged in outdoor jobs.

**Figure 1:**
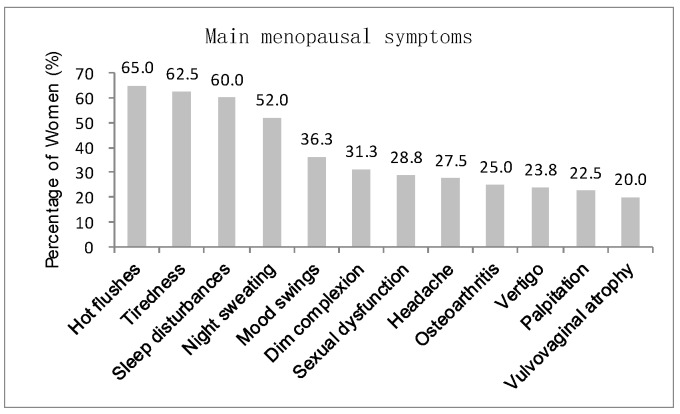
Main menopausal symptoms of participants.

**Figure 2:**
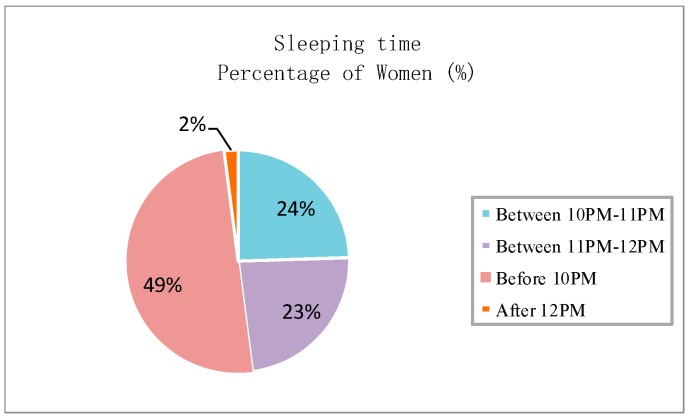
Sleep time of participants.

All women participants were asked to recognize the menopausal symptoms that they experience from a list of 19 symptoms referring to [22]. Figure 1 indicates that a high number of people (above 20%) selected 12 symptoms, including hot flashes, tiredness, sleep disturbances, night sweating, mood swings, dim complexion, sexual dysfunction, headache, osteoarthritis, vertigo, palpitation, and vulvovaginal atrophy. The other symptoms (below 20%) are heavy periods, poor concentration, lowered confidence, anxiety, tearfulness, clumsiness and weight gain. The most commonly-experienced symptoms among the participating women are hot flashes (65%), tiredness (62.5%), sleep disturbances (60%), and night sweating (52%). Figure 2 summarizes the sleeping time of the participants. In particular, 49% of them sleep before 10 PM; 24% and 23% sleep between 10 PM–11 PM and 11 PM–12 AM, respectively; merely 2% sleep after 12 AM. Therefore, most elderly women have a reasonable rest schedule, and the menopausal symptoms selected by the participants are slightly affected by an unhealthy rest style. This finding also verifies that hot flashes remain the most salient menopausal symptoms among the elderly women in southern China.

**Figure 3:**
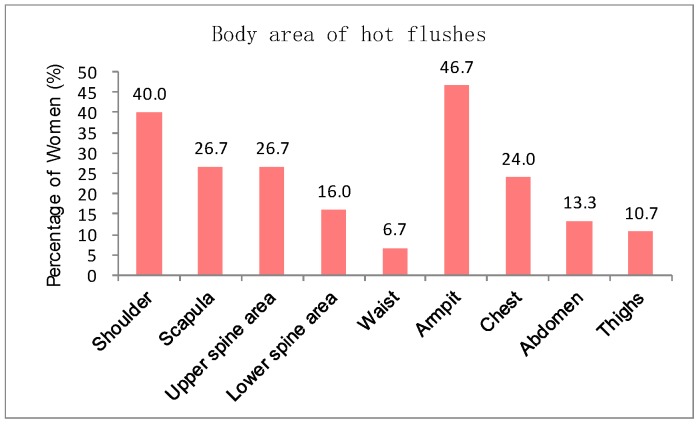
Body areas of hot flashes.

**Figure 4:**
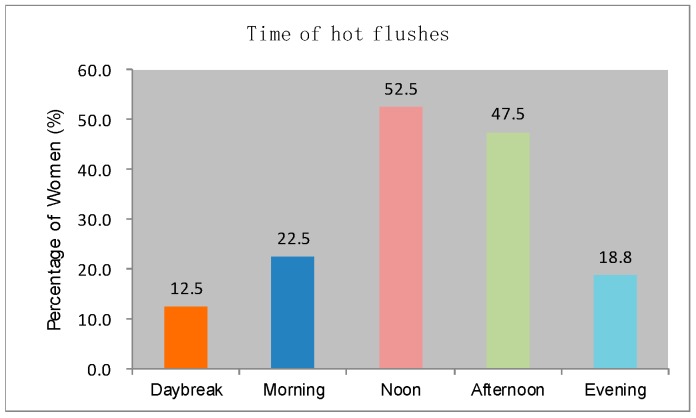
Occurrence time of hot flashes.

**Figure 5:**
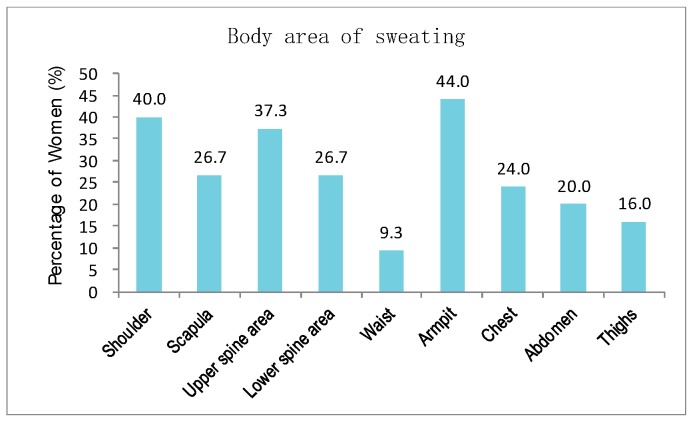
Body areas of sweating.

**Figure 6:**
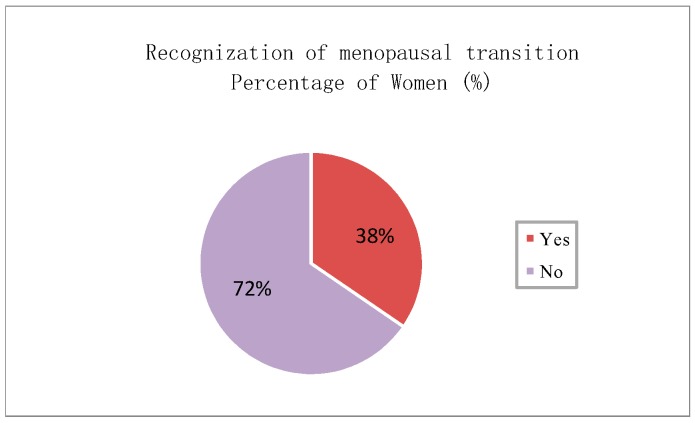
Awareness of menopausal transition.

Furthermore, the participants were asked to identify the body areas where hot flashes and sweating possibly occur. Figure 3 reveals that the hot flashes possibly occur on shoulder, scapula, upper spine area, lower spine area, waist, armpit, chest, abdomen, and thighs. The most salient areas in order are armpit, shoulder, scapula, upper spine area, chest, and abdomen. The participants experience hot flashes more frequently at noon and in the afternoon than at other time of the day (Figure 4). Figure 5 indicates that sweating, which is typically accompanied by hot flashes, possibly occurs on body parts. The armpit, shoulder, upper spine, lower spine area and chest areas have high percentages of tendency to sweat. A final question in the survey was designed to investigate whether the participants are aware that they are entering or experiencing menopausal transition. Figure 6 shows that merely 38% of the participants recognize this fact. Therefore, a great potential is evident to provide the elderly women with a convenient monitoring device to help them recognize the menopausal phase as early as possible. Thus, they can receive appropriate treatments in time.

## 3. Sensor-Based Smart Clothing Monitoring System

Survey analysis reveals that the hot flashes and accompanying sweating are vasomotor symptoms and salient among middle-aged women, which motivates the quantitative monitoring of physiological parameters associated with these symptoms. The physiological changes that accompany hot flashes are generally conceptualized as endocrinological results, which would be measured by hormone levels, body temperature, and skin conductance. However, the hormone profile exhibits great fluctuation for symptomatic and asymptomatic women. Sternal skin conductance, which is an indicator of sweat gland activity, remains controversial in literature for measuring the hot flash. Tataryn et al. (1981) found that sternal skin conductance has a strong correlation with subjective reports of hot flashes [27], whereas Carpenter et al. reported that the objective change in sternal skin conductance should not be used as a substitute measure of subjective hot flash intensity or distress [28]. Regardless of whether change in body temperature is measured on the skin or in the core, it does not particularly indicate hot flashes, but the rise in skin temperature most probably occurs following vasodilation and a subsequent increase in blood flow [29]. Freedman [30] pointed out that peripheral vasodilation proven by increased skin temperature occurs as hot flashes are experienced all over the body, including fingers, toes, cheek, forehead, forearm, upper arm, chest, abdomen, back, calf, and thigh. Skin temperature varies within a wider range than core temperature and has close relevance with the change of sweating and metabolic rates. Thus, measurement of skin temperature and relative humidity (another indicator for sweat gland activity) on multiple areas can compensate for the shortcomings of single physiological parameter and has strong indication for hot flashes. In practice, skin temperature has been widely used for clinical diagnosis because it is an easily detected physiological index that directly reflects whether the body is hot or ill [31]. Given these facts, we select multiple temperatures and relative humidity sensors to design a smart clothing system to help monitor women’s symptoms of hot flashes and consequently recognize menopause transition.

**Figure 7:**
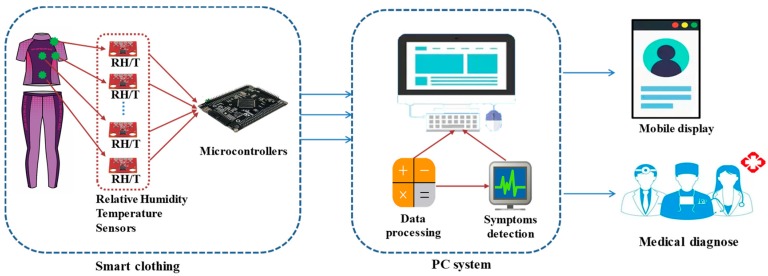
Sensor-based smart clothing for monitoring menopause transition.

Figure 7 illustrates the system of the sensor-based smart clothing for monitoring menopause transition. Smart clothing is designed with temperature and relative humidity sensors placed on different locations to measure skin temperature and relative humidity. All sensors are connected with a microcontroller, which collects sensor data and sends them to the PC system through wireless connection. The received data on the PC ender are processed to remove noise and drive menopausal detection algorithm, which outputs the detailed levels of menopausal symptoms. The monitoring results can be displayed on the mobile device for the user-friendly reading and obtaining of information.

**Figure 8:**
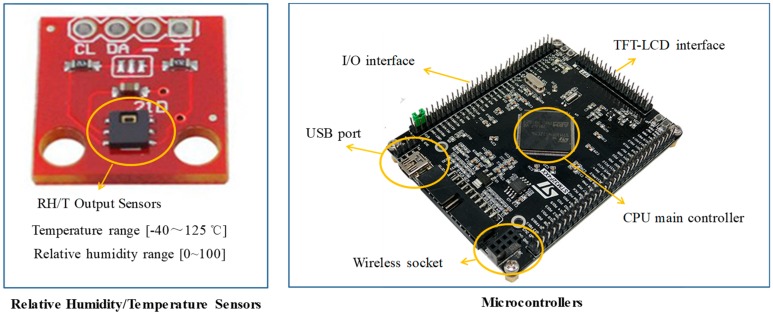
Sensors and microcontrollers used in smart clothing.

**Figure 9:**
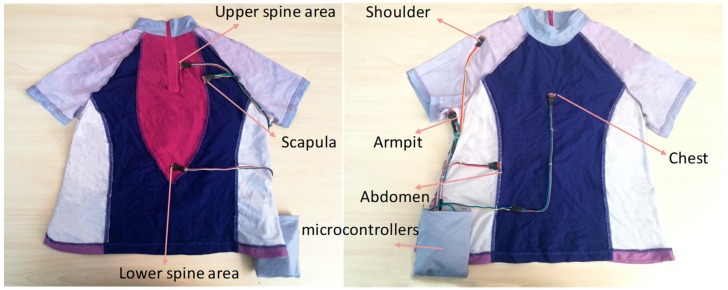
Smart clothing with sensor placement.

Wearable and attachable sensors have recently become popular for monitoring skin surface temperature and humidity for health purpose [32,33,34]. In this paper, we use a group of digital relative humidity sensors with temperature output (RH/T) that can thoroughly measure skin temperature and sweating rate by an entire module (Figure 8). The sensors provide calibrated and linearized signals in digital format and display direct interface with a microcontroller with the module for humidity and temperature digital outputs. Under 3.8 V supply voltage, temperature measurement ranges from −40 °C to 125 °C with ±0.1 °C accuracy between −5 °C to 50 °C and humidity measurement range is from 0 to 100% with ±2% accuracy. The placement of the sensor on the body location is highly device-specific and independent for different products given their various goals of monitoring [35]. The previous questionnaire reveals that the armpit, shoulder, scapula, upper spine area, and chest areas have high frequency for hot flashes, and armpit, shoulder, upper spine area, lower spine area, chest, and abdomen have high frequency of sweating. Thus, we place seven sensors on armpit, shoulder, scapula, upper spine area, lower spine area, chest, and abdomen. The sensors are implanted on the clothing by sewing on the fabric’s inner surface (Figure 9). We use a microcontroller to connect all the sensors and send the measured data from sensors to the computer via wireless communication.

## 4. Data Collection and Process

When worn on the human body, the RH/T sensors implanted on the smart clothing measure the skin’s relative humidity and temperature signals with resolutions of 12- and 14-bit readings, respectively. The two last least significant bits in the signals are used for transmitting status information, in which Bit 1 indicates the measurement type of temperature or relative humidity through the value of 0 or 1. The signals are further converted into digital data with physical magnitude and transferred to the microcontroller unit (MPU) through the wire connection. The MPU temporally stores the received data in buffer memory and transmits them to the PC ender by establishing wireless communication. All RH/T sensors are connected to the MPU and transmit the measured data to the PC sequentially.

**Figure 10:**
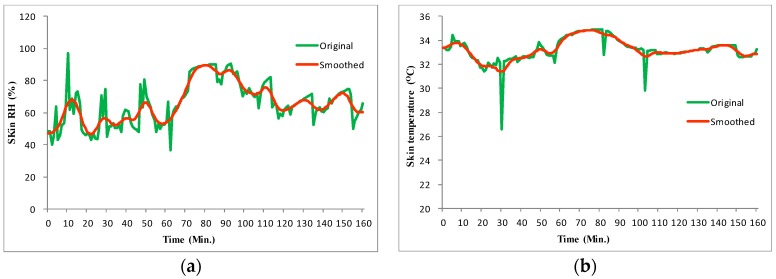
Measured data smoothing by moving average filter. (**a**) Smoothed skin relative humidity data, (**b**) Smoothed skin temperature data.

The computer distinguishes the received data into skin temperature and relative humidity data and resamples with intervals of 0.5 min. According to the number of the RH/T sensors, skin temperature and relative humidity data accompanied by time information are stored in different files. However, given the unstable contact between the skin and the sensors, the measured data exhibits considerable noise and vibration along with the distribution, which may generate abnormal data outliers or deviation errors in data, thus complicate the analysis of the trends of skin temperature and relative humidity change. Thus, we process these original data with moving average filter [36] that takes a set of input samples at a time, averages these samples, and produces a single output point to achieve refined data for driving hot flash detection algorithm in the next section. Figure 10a,b shows smoothed skin relative humidity data and temperature data respectively by using the moving average filter algorithm. It can be seen that the smoothed results have successfully removed the data outlier and meanwhile the possible deviation in data could be narrowed in the trend since the moving average filter algorithm takes the average between neighboring data.

## 5. Hot Flash Detection Algorithm

The analysis in Section 2 revealed that hot flash is the most salient symptom of menopause transition for women in Southern China. Thus, hot flashes experienced by women can be detected to assist the diagnosis of menopause transition. Hot flashes are commonly transient episodes described as sensations of intense heat accompanied by profuse sweating, followed by frequent shivering and palpitations [37]. A hot flash begins suddenly, and a surprising heat sensation spreads through the face and upper body, which is different from the hot sensation throughout the body that is experienced by normal people. The current understanding about the cause of hot flashes remains incomplete, and the sensation preceding the onset of a hot flash is unclear [38]. In general, thermoregulatory set points are slightly destabilized during the perimenopausal and postmenopausal stages, which alters the thermoregulatory response and leads to vasodilation. A hot flash has been gradually characterized by several measurable thermoregulatory parameters, such as body temperature, sweating and heart rate during the past decades. The measurement methods or tools could be particular to hot flashes when additional knowledge about the underlying phenomenon of such symptom is learned. Currently, skin conductance has been used by numerous researchers as the physiologic label for the objective measurement of menopausal hot flashes, but the monitoring results are generally inconsistent with subjective hot flashes, including the underdetection of subjective report [39]. Several research have measured finger temperature, which can rise from 1 to 7 °C during a hot flash, or skin temperature of multiple areas including finger and toe, forehead, chest, thigh, and upper arm [40]. However, the changes in skin temperature alone are inaccurate for measuring hot flashes [41,42]. The directions for improving the objective measurement of hot flashes are described as follows. (I) other stable physiologic parameters that are specific to hot flashes are identified, and promising technologies adapted to measure relevant data are explored; (II) measurement tools and detection algorithm are refined to improve the accuracy of evaluation, thereby potentially increasing understanding of hot flashes relevant to other phenomena.

Given these concerns, we propose a new trinity (frequency–duration–intensity) detection algorithm that combines skin temperature and relative humidity as physiological parameters for measuring menopausal hot flashes, in this paper. Skin relative humidity is another indicator for sweat gland activity, which can play a similar role of skin conductance for menopausal monitoring. Furthermore, skin relative humidity is easily measured with skin temperature by using the same sensor module, which enables the convenient implementation of the measurement. This indicator can integrate the advantages of the previous methods using either skin temperature or skin conductance. The input data of skin temperature and relative humidity for the detection algorithm are derived from multiple areas of the upper body, including armpit, shoulder, scapula, upper spine area, lower spine area, chest, and abdomen. The aggregation of the contributions of the indications enclosed in the measured data on the multiple areas is beneficial to improving the detection accuracy. Furthermore, this algorithm aims to output fine-grain control detection results with triple dimensions (frequency–duration–intensity) for menopausal hot flashes. This algorithm calculates the frequency of hot flashes occurring during the monitoring period, which is a common output in the previous methods, and works out the intensity and duration of hot flashes, which are two important characteristics of hot flashes. The triple-dimension monitoring results can offer an improved diagnosis for women’s menopause transition in terms of precision.

**Figure 11:**
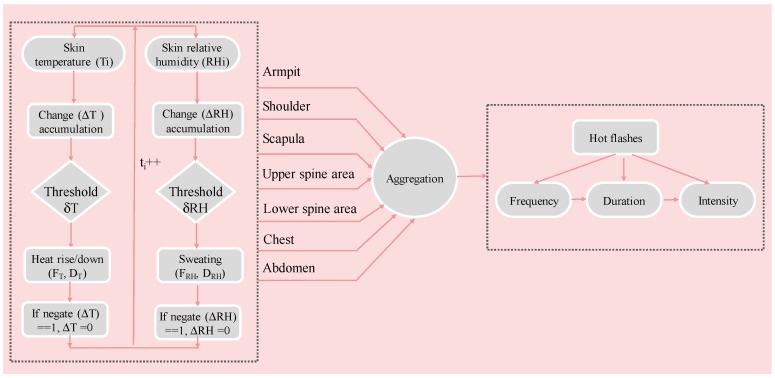
Physiological trinity detection algorithm for menopausal hot flashes.

Figure 11 demonstrates the trinity detection algorithm for menopausal hot flashes based on physiological parameters of skin temperature and humidity. Firstly, for each area of armpit, shoulder, scapula, upper spine area, lower spine area, chest and abdomen, measured skin temperature (T_i_) and relative humidity (RH_i_) are analyzed to calculate the frequency (F) and duration (D) of heat rise/down and sweating rise/down over time. The recognition of an increase or decrease of heat is triggered by whether the accumulated change (ΔT) during a period of continuous time step (t_i_ = 1) is beyond the threshold (δT). An event of sweating rise/down is also recognized by the accumulated change (ΔRH) that is triggered beyond the threshold (δRH). Kronenberg F (1987) indicates that the change in skin temperature during a hot flash can be as small as 0.5 °C or as much as 5.0 °C. The threshold for skin temperature (δT) is 0.5 °C, and the threshold for skin relative humidity (δT) is 5% [6]. When an event is recognized, the corresponding frequency of F_T_ or F_RH_ is added, and the duration (number of time steps) is recorded. Change accumulation ΔT or ΔRH is initialized to 0, and the event of heat or sweating rise/down is ended only when the trend of rise or down is changed, which can be evaluated by the operation of negation.

When the analysis of skin temperature and relative humidity data on all seven body areas during monitoring time is finished, the frequency and duration information of heat rise/down and sweating rise/down of each area are further aggregated to detect the event of hot flashes. Physiological criterion for a hot flash is designed as the concurrent occurrence of heat and sweating rise within a short period, which is set as 3 min, to overcome the limitation that the skin temperature change is not specific to hot flashes. Sturdee et al. [43] pointed out that the flush is only experienced while the skin temperature is increasing, that means our rule focusing on the heat rise in hot flash makes sense. The effect of sweating evaporation during measurement has already reflected on the skin temperature and skin humidity since these physical phenomena are coupled in practice.

The duration of a hot flash is calculated as the difference between beginning time and ending time of each hot flash. The beginning time of a hot flash is defined as the time when heat rise initially occurs because the heat generation should be earlier than the sweating on the skin. The ending time of a hot flash is defined as the ending time of an event of heat rise due to the sweating evaporation. The hot flashes recognized on all individual areas are aggregated within a period of time specified by the user, particularly 30 min. Skin temperature of many women generally drops when the skin has sweating effect, but several women may undergo prolonged hot flashes that last up to 60 min [4]; thus, we can distinguish the type of hot flashes as discrete or continuous. In the type of discrete hot flashes, the event of hot flash has no overlap between each other and the frequency may be noticeable, while in the type of continuous hot flash, there is only one hot flash but it has continuous long duration. Thus, as the frequencies on a single area cannot precisely reflect the symptom of hot flashes, it is wise to aggregate the occurrence of hot flashes on all the body areas to offer a more accurate indicators (defined as intensity). Table 1 lists the detailed calculation methods for the attributes of frequency, duration and intensity in aggregation.

## 6. Hot Flash Monitoring Experiment

**Figure 12:**
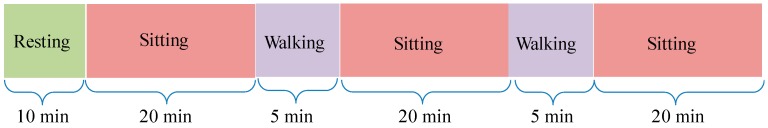
Activities of six sections in the experiments.

We designed and conducted a set of hot flash monitoring experiments to validate the hot flash detection algorithm by involving eight women aged 40–60 years. All participants had no evidence of medical or psychiatric disorders. Three have been experiencing hot flashes for 2–3 years and have not previously received estrogen therapy. Detailed physical information about the 8 participants is shown in Table 2. Prior to the experiment, all of them were instructed to stop all medications (if any) one week before and not to eat food or drink liquid (except water) at least 2 h before the experiment. The experiment time was chosen in the afternoon between 3:00 PM–5:00 PM, which is the time within a day when the highest frequency of menopausal hot flashes occur. The participants were allowed to rest for 30 min to reach equilibrium before any data collection. If the subject experienced hot flashes during this time, they would be asked to continue resting until they reach equilibrium. Each subject participated in six sessions including resting, sitting and walking activities to imitate the daily life of women, and the total time was 80 min (Figure 12). The experiment was conducted indoor with neutral thermal conditions, that is, air temperature of (25 ± 0.2) °C, air relative humidity of (45 ± 5)% and wind speed of < 0.1 m/s.

**Figure 13:**
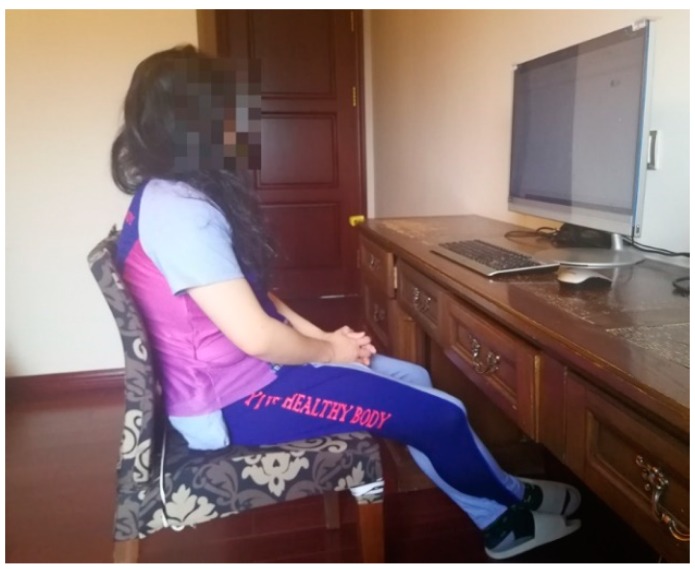
Data measurement scenario of women participants wearing smart clothing.

During the experiment, the subject wore the smart clothing, while the RH/T sensors on the clothing continuously measured skin relative humidity and temperature data of armpit, shoulder, scapula, upper spine area, lower spine area, chest, and abdomen. The PC ender received the measured skin temperature and relative humidity and separately stored them at 0.5 min intervals with the subject ID. The data files were refined and input for analysis of the hot flash detection algorithm. Figure 13 illustrates the scenario of women measuring the data in the experiment.

## 7. Experimental Results

**Figure 14:**
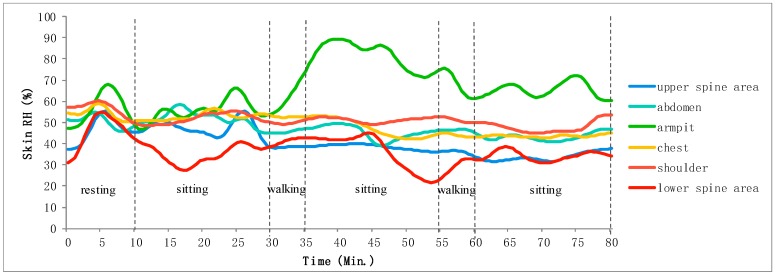
Skin relative humidity data of P1 during monitored activities.

**Figure 15:**
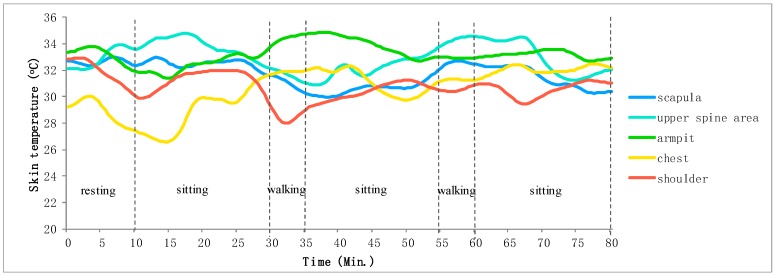
Skin temperature data of P1 during monitored activities.

All collected measurement data from the eight women subjects were refined by initially removing the outlier points. As mentioned above, skin temperature data from armpit, shoulder, scapula, upper spine area and chest could infer hot flash well, and skin relative humidity data from armpit, shoulder, upper spine area, lower spine area, chest and abdomen indicate sweat amount. They can be demonstrated by graphic presentation for convenient observation. Figure 14 indicates the skin relative humidity data of P1 (P1–P8 represent the eight subjects) and marks the corresponding activities during data measurement. Figure 15 reports the skin temperature of P1 during the same activities. Skin relative humidity and temperature data of different body areas exhibit different rises and falls during the same activity.

**Figure 16:**
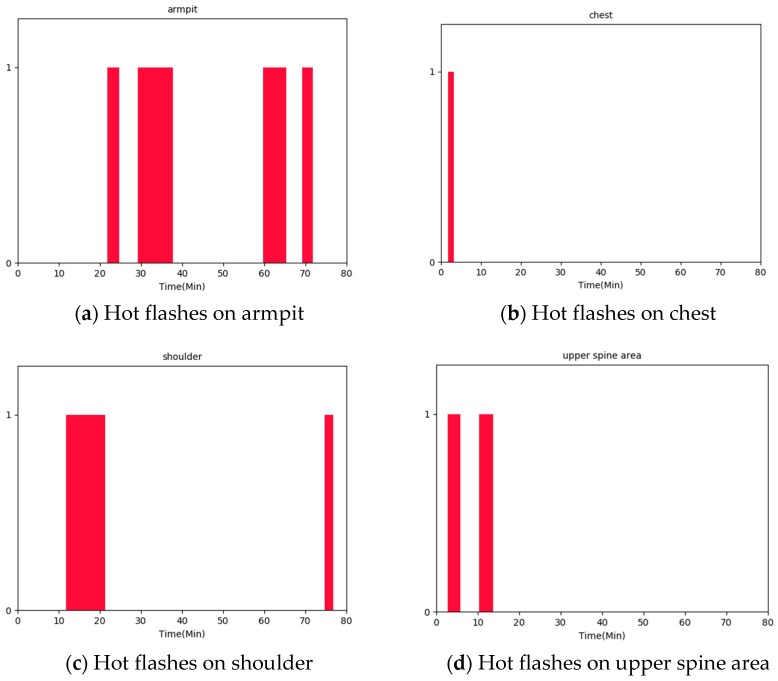
Hot flashes recognized on body areas of (**a**) armpit, (**b**) chest, (**c**) shoulder and (**d**) upper spine area of P1.

Given these processed skin relative humidity and temperature data, the event of a hot flash can be recognized by accounting for the concurrent occurrence of heat and sweating change within 3 min. In addition, the duration of a hot flash can be calculated by determining the beginning time when the skin temperature starts to rise and the ending time when skin temperature stops falling following skin temperature rising. The same body areas in monitored skin relative humidity and temperature include armpit, chest, shoulder, and upper spine area. Thus, hot flashes are recognized on these four body areas. Figure 16a–d reveals the recognized hot flashes of P1 on armpit, chest, shoulder and upper spine area respectively from the measured skin relative humidity and temperature. The hot bars in the charts mean the occurrences of hot flash, and the widths of the bars represent the durations of hot flashes. Such as, on armpit, there are four times of hot flashes happened with 2 min, 8 min, 6 min, and 2 min duration respectively. On the areas of armpit, chest, shoulder and upper spine area, the frequency of hot flash is highest on the armpit, which is followed by that on shoulder and upper spine area. By contrast, the least frequency of hot flash is on the chest. These results are consistent with the biological mechanisms of human beings showing that the armpit is most sensitive for generating heat and sweat in all body areas. From Figure 16, it can be seen that our method can precisely recognize both the occurrence and duration of each event of hot flashes detail to different body areas, which provides a significant overview of the symptoms of hot flashes for monitored women. Importantly, this recognized information offers the basis to predict the severity of women’s menopausal transition.

**Figure 17:**
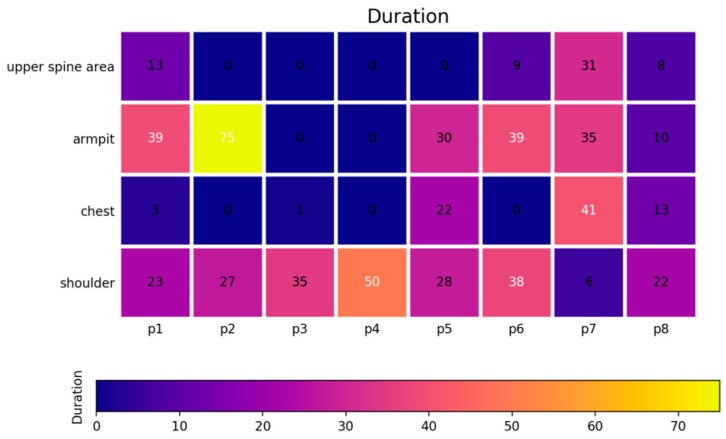
Durations (min) of hot flashes of four body areas among all eight women subjects.

**Figure 18:**
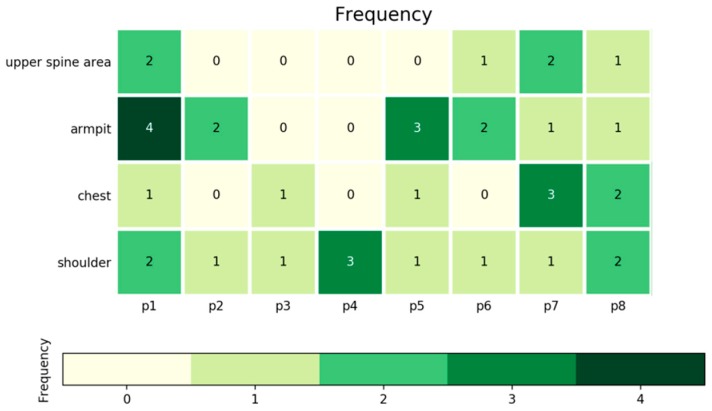
Frequencies of hot flashes of four body areas among all eight women subjects.

**Figure 19:**
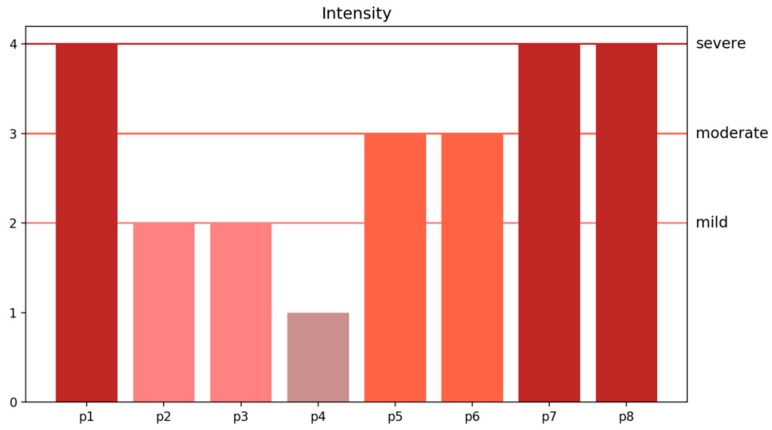
Intensities of hot flashes of four body areas among all eight women subjects.

Given the recognition of hot flashes on these four body areas, the duration, frequency, and intensity of hot flashes can further be analyzed and thus provide beneficial evidence for diagnosing menopause transition. Figure 17 and Figure 18 present the accounted durations and frequencies of hot flashes on the armpit, chest, shoulder, and upper spine area among the eight women subjects. In Figure 17, the number shown in each grid means the duration with the unit of minutes, and in Figure 18, it represents the times for frequency. Meanwhile, the grids in these charts are visualized with mapping colors according to the color bars for more easy understanding. It can be seen that the P1, P7, and P8 subjects have more frequent and higher duration of hot flashes than others. Several discrete data, including duration of hot flashes on the armpit of P2, are much higher than others. However, this result does not necessarily mean that the subject experienced severe hot flashes. Figure 17 and Figure 18 have important practical significance that they avoid the tedious record and accounting during the manual measurement which is easy to have errors due to artificial operations. These pictures can directly and precisely deliver the duration and frequency of hot flashes on the whole body in an effective way, thus it is able to make particular treatment for the body areas which have heavy hot flashes. Furthermore, the summary of duration and frequency on the body areas can be used to evaluate the intensity of the symptom of hot flushes, which indicates the severity of the symptoms of menopause. Figure 19 reports the evaluation of the intensities of hot flashes among the eight women subjects according to the definition of intensity of a hot flash listed in Table 1. Subjects P1, P7, and P8 experienced severe hot flash syndrome. From Figure 19, the doctors and clients thus have an overall evaluation on the severity of the symptoms of menopause and then can develop appropriate healthcare plan. The predicted duration, frequency and intensity of hot flashes in Figure 17, Figure 18 and Figure 19 are consistent with the self-reported data of the eight subjects during the experiment.

## 8. Discussion and Conclusions

The absence of a convenient testing method to recognize menopause, particularly for employed women, has been a dilemma for a long time. Current methods are based on symptom detection in a hospital, which merely capture the frequency or occurrence of hot flashes but fail to obtain the duration or intensity [2]. However, several indicators from physiological information, such as skin temperature, sweating, or hormone levels, may also be more useful than symptom detection. Our work hopefully addresses these gaps by providing a set of smart clothing that can be worn daily to monitor physiological parameters associated with hot flashes, which is the most evident symptom of menopause transition. Smart clothing is an ideal choice to detect health care problems through long-term monitoring, which relieves the necessity to go to a hospital frequently. The issues involved in the design of smart clothing monitoring system include (1) determining the sensor-measured data which have strong inference with the health care problem, (2) determining the area of clothing to place the sensors, and (3) designing algorithms to process data and calculate useful results for diagnosis. Our work addresses these issues and offers a sensor-based method to collect data and output duration, frequency and intensity of hot flashes.

This study identifies skin temperature and relative humidity as the appropriate physiological data for measuring and body areas for monitoring through a survey analysis of menopausal transition symptoms involving 100 employed women participants with different backgrounds. Using this preliminary information, a suit of smart clothing is designed by attaching a set of temperature and relative humidity sensors on armpit, shoulder, scapula, upper spine area, lower spine area, chest, and abdomen areas. All sensors are connected by a microcontroller, which sends the measured data to the PC. A new trinity (duration–frequency–intensity) detection algorithm, which takes the refined skin temperature and relative humidity data as inputs, is proposed for measuring menopausal hot flashes. The recognition of the event of a hot flash is defined as the concurrent occurrence of heat and sweating change within a specified duration, which overcomes the limitation that the skin temperature change is not specific to hot flashes. This algorithm based on the data analysis on multiple body areas outputs fine-grain detection results including duration, frequency and intensity for menopausal hot flashes, thereby providing more useful information compared with previous methods that merely calculated the frequency of hot flashes. These triple-dimension monitoring results offer an improved diagnosis for women’s menopause transition. The actual experiment involving eight women subjects demonstrates that the detection algorithms work well in recognizing hot flashes that occur on different body areas, subsequently figuring out the detail frequency–duration–intensity dimension data of hot flashes among individual subjects. This sensor-based smart clothing is promising for helping elderly women to conveniently monitor their menopause transition in their homes.

## Figures and Tables

**Table 1:** Calculation methods for the frequency–duration–intensity of hot flashes.

Hot Flash Attributes	Calculation Methods
Frequency	∑ni, sum of the number of hot flashes recognized on all monitored body areas
Duration	The duration of discrete hot flashes is same as that calculated on the individual area.
	The duration of a continuous hot flash, which exhibits a couple of intersected hot flashes, is the ending time of the last hot flash less the beginning time of the first one.
Intensity	Mild, if over 20% of monitored body areas has hot flashes
	Moderate, if over 50% of monitored body areas has hot flashes
	Severe, if over 80% of monitored body areas has hot flashes

**Table 2:** Brief information of participants.

NO.	Age (y)	Height (cm)	Weight (kg)	Occupation
P1	51	168	56	Indoor job
P2	52	152	50	Indoor job
P3	60	164	62.5	Outdoor job
P4	47	163	78	Indoor job
P5	55	155	54	Indoor job
P6	58	166	60	Indoor job
P7	48	163	65	Indoor job
P8	53	158	75	Indoor job
